# Early Emerging Gradients in Children's Eye Movement Times Across Levels of Household Resources

**DOI:** 10.1111/desc.70058

**Published:** 2025-08-12

**Authors:** Jukka M. Leppänen, Juha Pyykkö, Denise Evans, Lezanie Coetzee, Günther Fink, Aisha K. Yousafzai, David H. Hamer, Doug Parkerson, Peter C. Rockers

**Affiliations:** ^1^ Department of Psychology and Speech‐Language Pathology University of Turku Turku Finland; ^2^ Department of Global Health Boston University Boston Massachusetts USA; ^3^ Health Economics and Epidemiology Research Office, Faculty of Health Sciences, Department of Internal Medicine, School of Clinical Medicine University of the Witwatersrand Johannesburg South Africa; ^4^ Swiss Tropical and Public Health Institute University of Basel Allschwil Switzerland; ^5^ Department of Global Health and Population Harvard T.H. Chan School of Public Health Boston Massachusetts USA; ^6^ Section of Infectious Diseases, Department of Medicine Boston University School of Medicine Boston Massachusetts USA; ^7^ Innovations for Poverty Action New York New York USA

**Keywords:** early environment, eye tracking, infant, poverty, reaction time, saccade, visual function

## Abstract

Studies in low‐resource settings suggest that multiple aspects of early childhood development are sensitive to the relative poverty of a child's environment. We examined whether direct, quantitative measures of early developing cognitive functions show a similar association with relative poverty. Eye movement latencies were recorded in children at 7, 17, and 36 months in rural South Africa (*N* = 374). The latency to respond to the appearance of visual objects was inversely associated with a proxy measure of the child's socioeconomic environment (household asset ownership), with longer latencies observed in children from households with less asset‐based resources. This gradient was detectable at 7 months, increased between 7 and 36 months, was not explained by differences in prior exposure to screens, and generalized to the latency of eye movements towards socially cued objects (i.e., pointing gestures that directed the child's attention to a specific object). Physical growth (height‐for‐age) and developmental milestone scores were lower in children from households with less resources. The results were replicated in an analysis of independent data from 31‐month‐old children collected in Zambia (*N* = 270). The results provide novel evidence for early emerging gradients in behaviors that are universal and mechanistically involved in learning and skill formation.

**Summary:**

- Eye tracking was used to assess whether the early development of elementary visual behaviors is associated with the relative poverty of the environment in low‐resource settings.
- Eye movement latencies to the onset of visual stimuli were longer in children from relatively poorer environments, with suggestive evidence for a steepening of this gradient over early childhood.
- A similar gradient across poverty levels was seen in eye movement latencies to dynamic social spatial cues (gaze and hand gestures)
- This study provides novel, quantitative evidence for very early‐emerging gradients in behaviors that are essential for adaptive functioning and learning across all environments.

## 1 Introduction

Early‐life interactions with the environment are critical to the development of human behavior and its variations across individuals and groups. Differences in brain development and skill formation may arise from inequalities in environments and experiences (Johnson et al. 2016; Black et al. 2017; Walker et al. 2007;;2011), with multiple studies suggesting that children's developmental outcomes become less favorable as the poverty of the child's rearing environment increases (Ajayi et al. 2020; Donald et al. 2019; Drago et al. 2020; McCoy et al. 2016; Fernald et al. 2012; Rubio‐Codina et al. 2015; Amso and Lynn 2017; Wang and Fitzpatrick 2019). This gradient in early outcomes has so far been observed in measures of physical growth and parent‐ or expert‐made qualitative assessments of developmental milestone achievement (McCoy et al. 2016; Fernald et al. 2012; Rubio‐Codina et al. 2015). However, very little is known about whether a similar gradient is observed in more direct and objective measures of behavioral development.

Among the skills that can be directly quantified and are mechanistically involved in early learning across all environments are elementary visual behaviors, such as the (saccadic) eye movements that align the area of sharp foveal vision with external targets. These eye movements are present at birth (Valenza et al. 2015), undergo predictable age‐related improvements in timing during the first years of life (Alahyane et al. 2016; Hunnius et al. 2006), and are integral in early, socially guided learning across cultures (Hernik and Broesch 2019; Hessels 2020). Because saccadic eye movements can be assessed in an automated manner in children across ages and rearing environments (Hernik and Broesch 2019; Pyykkö et al. 2019; Leppänen et al. 2022; Boivin et al. 2017; Chhaya et al. 2018; Familiar‐Lopez et al. 2023; Forssman et al. 2017; Katus et al. 2019; Prado et al. 2020; Pyykkö et al. 2020; Leppänen et al. 2022), this measure may have utility in objectively documenting the emergence of individual differences in cognition across rearing environments.

Preliminary data suggest that, similar to other early developing skills, the latency of saccadic eye movements may co‐vary with socioeconomic factors. Direct comparisons have revealed a significant difference in mean saccadic reaction times (SRTs) among 9‐month‐old infants between a low‐ and a high‐income country (Forssman et al. 2017). Within high‐income settings, SRTs have been inversely associated with family income, with shorter SRTs observed at higher income levels (Conejero and Rueda 2018). However, a similar association has not been observed in relation to parental education (Siqueiros Sanchez et al. 2021).

To our knowledge, no studies have examined whether the latency of eye movements is associated with the relative poverty of the home environment within low‐income settings, as is the case for other aspects of early development in these contexts. Studies focusing on low‐resource settings are important, as early childhood development may be influenced by different factors in these environments compared to the more extensively studied high‐resource settings (Amso and Lynn 2017). While not necessarily detrimental to development on their own, low‐resource environments tend to be associated with a higher prevalence of experiences that can be harmful, including malnutrition, stress, household crowding, and limited cognitive stimulation (Amso and Lynn 2017). It is also possible that early cognitive development is relatively more susceptible to the influence of environmental factors in low‐resource environments (Tucker‐Drob et al. 2011), such as effects that arise from reduced myelination and slower neural transmission due to malnutrition (Naila et al. 2025; Ursache et al. 2016), because children in these settings may lack access to compensatory experiences that might otherwise mitigate such effects (Ursache et al. 2016). Collectively, these considerations underscore the theoretical and practical importance of understanding whether and how early cognitive development is influenced by various risk factors within the specific context of low‐resource settings, rather than merely extrapolating from findings obtained in high‐resource settings.

To obtain objective data on variations in early developing cognitive functions in a low‐resource environment, we conducted repeated assessments of SRTs in a cohort of children in rural South Africa at child age 7, 17, and 36 months. Our primary aim was to examine whether and at what age SRTs, as a direct measure of elementary visual behavior in infants, begin to show a socioeconomic gradient—that is, a negative correlation with a proxy measure of the resources of the rearing environment (household assets) (Fernald et al. 2012). In further analyses, we examined whether this gradient is seen when potential effects of the specific experience of viewing screens is controlled, because screen ownership may covary with poverty, and the speed of eye movements can vary based on the history of screen use (Portugal et al. 2021). This study was based on secondary analyses of data from an intervention study (Rockers et al. 2023) and was not preregistered. The replicability of the results was investigated by analyzing data from an independent study with 31‐month‐old children in Zambia.

## 2 Methods

### 2.1 Participants

Data for the primary analyses were obtained from a study conducted in Greater Tzaneen sub‐district, Limpopo Province, South Africa (Rockers et al. 2023). The estimated rates of multidimensional poverty among children under 4 years—defined as inadequate sanitation, nutrition, protection, health care, early childhood education, and housing conditions UNICEF (UNICEF)—are high in South Africa (59.9%), and disproportionally high in the rural areas of South Africa where this study was conducted (83%). Replication data were obtained from a study conducted in Lusaka, Zambia Fink et al. (2023), where child poverty is also high, with an estimated 86.8% of children in rural and 41.8% of children living in urban areas suffering from two or more dimensions of well‐being (Zambia Statistics Agency 2023). Data for the current sample were collected in urban and peri‐urban areas in the Lusaka district in Zambia.

#### 2.1.1 South Africa Cohort

Participants in the South Africa cohort were a subsample of caregiver–child dyads enrolled in a cluster‐randomized controlled trial that was designed to evaluate the impact of a package of early childhood interventions in Mopani District (SANCTR registration number: PACTR201710002683810) (Rockers et al. 2023). Dyads that were enrolled in the trial and resided in Greater Tzaneen sub‐district were invited to participate in a substudy that involved the collection of EEG and eye tracking data. Further inclusion criteria were as follows: (1) child born between December 15, 2017 and March 15, 2018; (2) primary caregiver at least 18 years old at enrollment, and (3) birthweight > 2500 g, based on data extracted from well‐child records provided to caregivers in South Africa. Of the 384 dyads who participated in at least one eye tracking assessment, 374 were retained in analyses of SRTs based on meeting the inclusion criteria and having sufficient data at one or more age points (205 participated at all three age points; 89 had valid data from all age points). The criteria for valid SRT data are explained below.

#### 2.1.2 Zambia Cohort

Participants in the Zambia cohort were a subsample of approximately 2300 caregiver‐child dyads enrolled in a cluster‐randomized factorial trial aimed at investigating the effects of a growth chart intervention and nutritional supplements on child growth (clinicatrials.gov registration number: NCT05120427). This trial was conducted across 280 communities (clusters) in the Luapula, Lusaka, and Southern provinces of Zambia. Dyads participating in the main trial and residing in communities within Lusaka district were invited to partake in a substudy involving the assessment of SRTs using eye tracking. Recruitment occurred through the following two steps: (i) a random selection of 90 enumeration areas within the study districts, based on the 2020 census sampling frame provided by the Central Statistical Office of Zambia (CSO), and (ii) home visits to all households in the selected areas, to compile a roster of households with infants aged between 7 and 14 months. Subsequently, eligible dyads were visited by trained study personnel, who provided comprehensive explanations of the study to caregivers and enrolled them upon obtaining their consent. Dyads with plans to relocate from the study area within 12 months of the recruitment period were excluded from the study.

Of the 316 children who passed the inclusion criteria and participated in the eye tracking assessment after the intervention (mean age 30.8 months), 275 (87%) were retained in analyses of SRTs based on meeting the criterion of sufficient data, as explained below. A subsample of the children (*n* = 14) was from the same households.

### 2.2 Ethics  Statement

Ethical approvals for the study in South Africa were received from Health Economics and Epidemiology Research Office (HE2RO), University of the Witwatersrand, South Africa and Ethics Committee of the Tampere Region, University of Tampere, Finland. Ethical approvals for the study in Zambia were received from the Ethics Committee Northwest Switzerland (EKNZ—AO2021‐00016) and the University of Zambia Biomedical Research Ethics Committee (1411‐2020). Informed consent was obtained from all caregivers of participants with a private oral explanation of the procedure, risks, and benefits. All caregivers signed or thumb‐printed the consent form on behalf of their children.

### 2.3 Eye‐Tracking Measures

Families participating in the study were invited to visit a centrally located laboratory when children reached 7, 17, and 36 months of age in South Africa, or during the intervention's endline visit around the mean age of 31 months in Zambia. During the lab visit in South Africa, the children were administered resting‐state EEG assessments (∼15 min) before eye‐tracking assessments (∼10 min). EEG assessment was not part of the protocol in Zambia.

#### 2.3.1 Assessments

**FIGURE 1:**
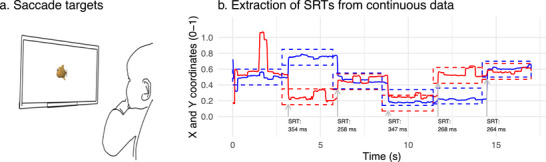
Measurement of eye movements. (a) Saccade targets were sequentially presented on randomly chosen locations on a computer screen, with a 9
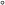
–14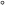 spacing between two targets. (b) Recorded *X*‐ and *Y*‐coordinates of gaze (blue and red lines) and target locations (blue and red rectangles) for one observer and sequence. SRT was defined as the time interval from the onset of the target to the first entry of the gaze into the area of the target (first target in the center was not used in the analysis). SRTs showed the expected levels of within‐ and cross‐visit stability (Supporting results).

Eye‐tracking measurements were conducted in a dedicated room, which was partitioned into separate spaces for the data collector and the participant (Supporting Methods). The caregiver held the child in a forward‐facing baby carrier or in their lap and were seated so that the child's eyes were at a ∼60‐cm distance from the eye tracker (Tobii X3‐120 or Tobii Pro Fusion) and at an optimal height in relation to the tracking “box” of the eye tracker (Figure 1). To ensure that the child's and not the caregiver's eyes were tracked, the caregiver was instructed to turn their head and eyes to the side (∼90º from the screen) and to avoid looking at the screen. The test consisted of repeated 3–4‐min sessions. Each session was composed of (1) calibration targets, (2) saccade targets, and (3) videos depicting social scenes. Details of these stimuli are provided in Supporting Methods. Calibration targets, saccade targets, and naturalistic videos of social scenes were presented in alternating sequence to minimize the monotony of the test sessions.

The first saccade target in the center of the screen was excluded from the analysis as the position of the gaze at the onset of the target and, consequently, the starting position of the first saccade was not standardized. Following the first target, children in South Africa saw five new targets per block across eight blocks (totaling 40 trials), while children in Zambia saw 11 new targets per block across six blocks (totaling 66 trials). In South Africa, four short and four long videos were presented between the saccade target blocks. In Zambia, six videos were presented.

The data collector monitored the testing and the child's position through online visualization of the tracked position of the child's head/eyes with respect to the optimal tracking position. If the eye tracking system lost contact with the child's eyes or the child became restless, inattentive, or fussy during the assessment, the data collector administered a break in the testing and performed required adjustments (e.g., adjusted the caregiver's and the child's position).

#### 2.3.2 Measures

The primary outcome was the latency of saccadic eye movement responses (SRT) to the onset of visual objects on a computer screen. Secondary (exploratory) analyses examined the latency to respond towards socially cued objects.

#### 2.3.3 Latency of Saccadic Eye Movement Responses

In South Africa, saccade targets were colored animated cartoon drawings of objects (e.g., bird, mouse, face, fish, pig, or soccer ball, size $∼5.7^{\circ }$ × 5.7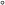), presented one at a time in randomly chosen on‐screen locations with a 10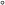 distance between two subsequent targets. In Zambia, saccade targets were sinusoidal grating stimuli with a Gaussian mask (size $∼5.0^{\circ }$ × 5.0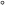). After saccade, an animated cartoon drawings of objects similar to those used in the South Africa study were superimposed on some of the gratings to maintain the child's interest in the task.

Following the procedure described in Leppänen et al. (2022), SRTs was defined as the time interval from the onset of the saccade target to the first entry of the gaze into the area of the saccade target (Figure 1). SRTs were extracted from data that had been filtered with a 15‐sample (128 ms) median filter to remove abrupt spikes, and in which the *xy*‐coordinates for the two eyes had been merged by averaging (if both eyes returned a valid data point), or by using the data of one valid eye. A 0.9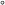 margin was added to the area of the target to accommodate calibration errors.

The extracted SRTs were regarded as valid and retained in the analyses if the following further inclusion criteria were met: (1) the starting position of the saccade was standardized so that the gaze moved from the area of the previous saccade target to the area of the new target (i.e., the gaze was within the area of the previous target for the entire time interval preceding the saccade, except a 50‐ms transition period); (2) the period from the onset of the new target to the registration of the saccade did not have consecutive missing samples exceeding 100 ms; (3) the gaze entry to the target area was not preceded by a missing sample (i.e., the exact point of entry was known within the limits of the eye tracking sampling frequency); (4) the SRT fell inside the commonly used expected time window starting 100 ms after target onset and ending 1000 ms after target onset. SRTs identified as outliers (2.5 SD from log‐transformed grand average SRT) were excluded from statistical analyses, which excluded 1.2%–2.7% of all SRTs on the three visits.

An additional post hoc analysis was conducted to verify whether the use of both binocular data (when tracking of both eyes was valid) and monocular data (when only one eye was tracked) might have resulted in false SRTs due to transitions between monocular and binocular tracking. These analyses showed that gaze estimation based on one eye alone was rare in our primary South Africa dataset (0.6% of the data that passed other quality checks), and there were no cases in which a transition between one‐ and two‐eye tracking (or vice versa) was associated with a false SRT (a shift in gaze between areas of interest).

Participants with ≥10 valid SRTs were retained in the analyses. Inclusion criteria used in the analyses were set a priori and identical to those used in the previous study (Leppänen et al. 2022).

#### 2.3.4 Latency of Eye Movements Towards Socially Cued Objects

Of the videos depicting social scenes in South Africa, a subset presented during the 36‐month visit included social cues with a clear spatially isolated target. The three videos with these characteristics were used in the analyses. The videos showed a frontal view of an adult person who alternated her attention between the camera/child and pictures of objects. The videos began with the person speaking to the camera/child in the child's native language (Tsonga or Sepedi), asking where a picture of a specific target object (e.g., a green bird) was. The person then pointed at the pictures one at a time, with the target object being the last one pointed at. The videos included four to seven pointing gestures (14 in total for the three videos in Tsonga, and 16 for the three videos in Sepedi), each with an identifiable starting point (i.e., the moment when the person in the video began to look at and point at one of the pictures shown) and target object. The duration of the videos ranged from 12 to 47 s.

The videos used in the Zambia study were similar to those from South Africa, showing a Nyanja‐speaking person who alternated her attention between the camera/child and various objects or spatial locations. In the videos, the person engaged in different activities involving shifts in her attentional focus, including searching for a target object (a ball) in three different locations, hiding and then searching for an object under one of two covered areas, and pointing at and describing drawings of different animals on the wall, one at a time. The duration of the videos ranged from 29 to 80 s, and the videos included two to nine events (23 in total) that met the requirement of a clearly identifiable shift in the person's attentional focus from the camera or one object to another, marked by head and/or eye movements. These moments were associated with a clear increase in looking at the targeted object (Supporting results), and were, hence suitable for the analysis of latencies.

Eye movements to social cues were analyzed using the same approach as that used in the SRT analyses. The data were segmented into 1‐s intervals starting at the onset of the social spatial cue. These segments were then preprocessed to exclude instances where (1) the child's gaze was not outside the area of the target object at the onset of the cue, (2) the period from the onset of the social cue to the registration of the first gaze point in the target object area had consecutive missing samples exceeding 100 ms, (3) the gaze entry into the target area was preceded by a missing sample (i.e., the exact point of entry was not known), and (4) the response latency to the social cue did not fall within the expected time window, starting 100 ms after cue onset and ending 1000 ms after cue onset. Responses identified as outliers (2.5 SD from log‐transformed grand average latency) were excluded from the statistical analyses (1.1% for South Africa and 3% for Zambia). Participants with three or more valid latencies were retained in the analyses.

### 2.4 Other Measures

#### 2.4.1 Height‐for‐Age

Anthropometric data were obtained during the 7‐, 17‐, and 36‐month visits in South Africa or at enrollment (mean age 5 months) and endline (31 months) in Zambia. The measures were transformed into *z* scores using WHO Child Growth Standards (Anthro V 3.1, WHO, Geneva). The height‐for‐age *Z* (HAZ)–score was used as a measure of chronic nutritional status of the child.

#### 2.4.2 Developmental Assessments

In South Africa, caregivers were asked to rate their child's early development using the Caregiver Reported Early Development Instrument (CREDI, McCoy et al. 2016) during the 7‐ and 17‐month visits. A standardized assessment of gross‐ and fine motor skills, language, and social‐emotional skills was administered by trained data collectors at the 36‐month visit with the Malawi Development Assessment Tool (MDAT) (Gladstone et al. 2010). In Zambia, milestone data were obtained by utilizing Global Scales for Early Development (GSED)—a culturally neutral and directly administered instrument for obtaining a global assessment of early childhood motor, cognitive, and language development (McCray et al. 2023). The data were available at the endline visit. Scaled total scores from all scales were used.

#### 2.4.3 Household Resources

In South Africa, data on household assets were collected as part of a household survey conducted upon enrollment into the study. The interviewer asked the caregiver whether the household had a given asset from a list of 29 assets. The list was drawn from the most recent South Africa National Income Dynamics Study, initiated by the South African Department of Planning, Monitoring, and Evaluation to track and understand poverty in South Africa. The assets reported included appliances (e.g., stove, refrigerator, washing machine); electronics (e.g., radio, television, computer); home floor, wall, and roof materials, vehicles (e.g., bicycle, automobile, boat); farming equipment (e.g., wheelbarrow, plough, tractor); livestock; and farmland. The asset ownership data were analyzed by principal component analysis (PCA) to construct a measure of household resources (Filmer and Pritchett 2001). A similar methodology was employed in Zambia, albeit with a shorter list of assets utilized in the interview questions and subsequent PCA. This abbreviated list included inquiries about ownership of television, mobile phone, bicycle, car, soap, as well questions concerning household electricity, floor material, roof material, water source, and toilet facilities.

Household asset ownership was positively associated with household size (number of occupants) in South Africa, Spearman rho (*n* = 326) = 0.21, *p* < 0.001, likely reflecting the fact that asset ownership was coded at the level of households, not families. This association was weaker in Zambia, Spearman rho (*n* = 313) = 0.05, n.s. Statistical analyses including household resources were adjusted for household size.

#### 2.4.4 Screen Time

In South Africa, screen time was assessed at the child age of 7 and 36 months. At 7 months, caregivers were asked to report whether the child watched or used a television, computer, or a DVD player with attached screen, and then indicate how often they have done so during the past 60 days using a 5‐point scale where 0 = “Never,” 1 = “Rarely” (1–4 times), 2 = “Sometimes” (5–8 times), 3 = “Often” (>8 times), and 4 = “On a daily basis”). At 36 months, the caregiver was asked to report the hours per day the child spends with computers, cell phones, handheld video games, and other electronic devices. A similar question was asked concerning the hours per day the child spends in front of TV. The caregivers were asked to give their answer in hours (0, 0.5, 1, 2…9) separately for weekdays and weekend days. Responses for weekdays and weekends were highly correlated and, therefore, averaged to construct measures for non‐TV and TV time at age 36 months. TV‐time from 7 and 36 months were used as an indicator of screen time in the analyses. In Zambia, parents' answers to a question on the time the child typically spends watching television separately for weekdays and weekends were summed and divided by 7 to obtain estimates of screen time (hours) per day.

### 2.5 Statistical Analysis

For descriptive analyses, differences in means were analyzed using Student's *t*‐test for normal variables and Wilcoxon test for non‐normal variables. Linear correlations between continuous measures were analyzed using Pearson *r* or Spearman rho. *p* values < 0.05 (two‐tailed) were regarded as statistically significant.

To assess whether eye movement measures were associated with household resources, the data were analyzed with a linear mixed model with the SRT as the response variable, visit, household assets, and visit × household assets as fixed effects, and random intercepts for child, stimulus block, and trial to account for the repeated measures nature of the data within and across the visits. The model was adjusted for child age at the visit (i.e., deviation from the visit's mean age) and household size (SRT ~ Visit × Assets + Age at visit + Household size + (1|Participant) + (1|Block:trial number)). In the analyses examining the association between eye movement measures and household resources when adjusting for the impact of covariates (screen time and HAZ), each covariate was added as a fixed effect in the model. A similar overall approach was used to analyze other early developmental outcomes (HAZ and parent ratings of development), except that covariate‐adjusted analyses were not performed for these outcomes. South Africa and Zambia cohort data were analyzed with similar approaches with the exception that Zambia analyses included varying distance of saccade targets as a covariate to account for the variations in distance as well as a nested random effect for family and child to account for the presence of same household members in the data. To prevent near singular fit for some of the Zambia models, random effects for trial and nested random effects for child were excluded. These changes did not lead to qualitative alterations in the results. The data for the current study were obtained from intervention studies. Preliminary analyses showed that the results were largely unaltered when intervention group was added to the models as a fixed effects covariate. Analyses and data visualizations were performed using R (version 4.0.3), ggplot2, and LME‐package (R Core Team 2020; Bates et al. 2015; Wickham 2016).

### 2.6 Data and Code Availability

The data that support the findings of this study are available on request from the corresponding author.

## 3 Results

Eye movements in children residing in Tzaneen, South Africa were assessed at 7, 17, and 36 months using remote, infrared eye tracking (Figure 1). Household resource assessment was based on the ownership of household assets (Table 1), aligning with the approach employed in previous studies to assess household resources within LMIC settings (Filmer and Pritchett 2001). Data for replication analyses were obtained from an independent cohort collected in the context of an intervention study in Lusaka district, Zambia. Children were seen twice in the study at the mean age of 5 months (pre‐intervention visit) and 31 months (post‐intervention visit).

**TABLE 1:** Characteristics of children in the primary (South Africa) and replication (Zambia) cohorts.

	South Africa				Zambia			
Variable	Mean	(SD)	Min	Max	Mean	(SD)	Min	Max
Caregiver age, yrs	31.8	(10.4)	18	83	27.6	(7.1)	15.0	55.0
Caregiver educ, yrs	10.3	(2.5)	0	16				
Assets, *n* ^a^	9.3	(3.3)	1	18	6.0	(1.4)	2.0	9.0
Child age at test, *m*								
Visit 1	7.6	(0.9)	6.6	11.2	5.2	(1.9)	2.0	10.3
Visit 2	16.4	(0.5)	15.6	18.0	30.8	(1.9)	27.0	35.0
Visit 3	36.8	(2.7)	30.8	41.1				
Height‐for‐Age *Z*‐score								
Visit 1	−0.4	(1.2)	−3.8	2.3	−0.8	(1.5)	−5.6	3.1
Visit 2	−0.8	(1.2)	−4.7	3.4	−2.0	(1.1)	−5.3	1.6
Visit 3	−0.3	(1.0)	−3.8	3.3				
Screen time (h/day)^b^	2.7	(1.8)	0.0	8.0	2.4	(2.3)	0.0	14.0

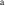
 Maximum number of assets was 29 in South Africa and 10 in Zambia. Screens were owned by 90% of households in South Africa and 78% in Zambia.

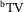
 time data from Visit 3 (90% of households in South Africa and 78% in Zambia owned TV screens).

### 3.1 Latency of Saccadic Eye Movement Responses (South Africa)

Of the participants assessed with eye tracking in South Africa 57% (Visit 1) to 86% (Visit 3) were retained in the analyses of SRTs based on the a prior inclusion criteria for the minimum number of valid trials (>9) and birth weight (>2.5 kg). Data retention rate (i.e., percentage of participants and number of valid SRT trials) improved by visit, as did the mean and the estimates of the within‐session stability of the SRT (Table 2). The success of SRT measurement (Figure 1), as evaluated by the the number of trials retained in the analysis after quality control, was not correlated with household resources (Supplementary Table S1).

**TABLE 2:** Descriptive data for Saccadic Reaction Times (SRT) by visit.

	South Africa			Zambia
Variable	Visit 1/7‐m.	Visit 2/17‐m.	Visit 3/36‐m.	Visit 2/30‐m.
*N* assessed	306	317	280	316
*N* retained	173	247	247	275
Valid trials (min, max)	18.7 (10, 39)	20.7 (10, 39)	25.1 (10, 38)	22.4 (10, 49)
Split‐half *r* [95% CI]	0.66 [0.56 0.74]	0.59 [0.5 0.66]	0.78 [0.72 0.82]	0.66 [0.59 0.72]
Mean SRT [95% CI]	395 [389 401]	385 [380 390]	337 [332 341]	339 [335 344]

SRTs had expected levels of stability within sessions (split‐half, odd‐even rs 0.59 to –0.78, Table 2) and across visits in South Africa (7 to –17 months, r*r*(114) = 0.49 [0.33, 0.61]; 17 to –36 months, r*r*(183) = 0.49 [0.37, 0.59]). These stability estimates are in line with prior studies in infants (Leppänen et al. 2022), which suggest that the speed of visual orienting in infants can have high variability on short (i.e., moment‐by‐moment or trial‐by‐trial) and longer (test‐–retest) time scales, whereas the split‐half and test‐–retest stability estimates are higher in adults (Leppänen et al. 2022; Bargary et al. 2017).

Data from South Africa showed that SRT improved slightly between the 7‐ and 17‐month visits and more clearly between the 17‐ and 36‐month visits (approx. 2.4 ms/month). The latter result is in line with those reported in prior studies reporting approx. 2 ms/month improvements (Alahyane et al. 2016), although direct comparisons of the values between ages and studies should be made with caution given differences in study procedures.

### 3.2 Latency of Saccadic Eye Movement Responses and Household Resources (South Africa)

**FIGURE 2:**
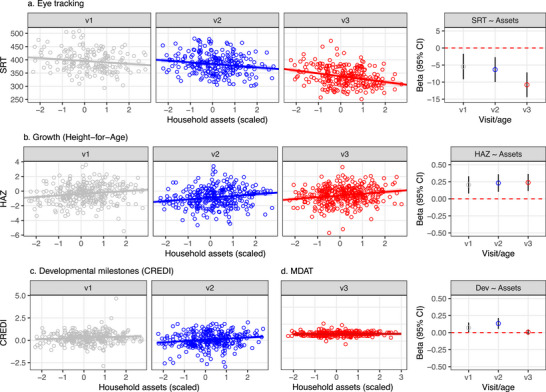
Early developmental outcomes by household resources (assets) and child age (visit) in the South Africa cohort. (a) Eye movement measures (SRT) by household assets and age. Observed and predicted values of mean SRTs based on household assets (left) and beta coefficients for household assets (right) from a linear mixed effect model with SRT as a dependent variable and household asset index as a predictor (model details are provided in Table S3). (b)–(d) Corresponding data for height‐for‐age *z*‐score (HAZ) and for developmental assessments obtained from parent ratings (CREDI, 7 and 17 months) and by using an assessment tool (MDAT, 36 months).

SRTs were longer in children from households with less resources, consistent with the existence of a socioeconomic gradient (β[95% CI] = −5.43 ms [−9.17, −1.69], Table S3). The gradient was observed at all ages, but there was also evidence that it became more pronounced between 7 and 36 months (β[95% CI] = −5.35 ms [−8.5, −2.2], Table S3 and Figure 2).

The association between SRT was further examined by adjusting the analyses for potential co‐variates (screen time and HAZ). Because the ability to detect and respond to stimuli on a computer screen may benefit from experience with computer and TV screens (Portugal et al. 2021), the observed gradient may be explained by differences in screen ownership and screen time across household with varying levels of resources. Data on screen time were collected at 7 and 36 months. Most households in the South Africa cohort owned a television (90%), but children in the households with less asset‐based resources spent less time watching TV at 7 months (*r*(170) = 0.29 [0.15, 0.43]) and at 36 months (*r*(245) = 0.16 [0.04, 0.28]). SRT was not associated with screen time at 7 months (*r*(170) = 0.04 [−0.11, 0.19]), but the predicted pattern was observed at 36 months, with longer SRTs in children who had spent less time watching TV (*r*(245) =−0.24 [−0.35, −0.12]). Critically, the association between SRT and household resources remained largely similar at 36 months when the analyses were adjusted for the significant effect of TV time at that age (β[95% CI] = −9.28 ms [−13.56, −5.00]). In the analysis with HAZ as a covariate, SRT was inversely associated with HAZ (β[95% CI] = −9.56 ms [−11.30, −7.79]), indicating that SRTs tended to be shorter in children with higher HAZ (i.e., better chronic nutritional status). The association between SRT and household resources remained after controlling for HAZ (β[95% CI] = −4.70 ms [−8.45, −0.96]).

### 3.3 Latency of Eye Movements Towards Socially Cued Objects (South Africa)

**FIGURE 3:**
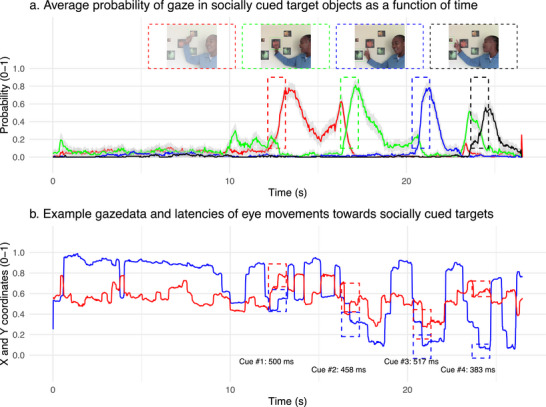
Latency of eye movements towards socially cued objects. (a) Mean probability (95% CI) of gaze within different object areas as a function of video time. The 1‐s period following a cue towards the object is highlighted with a colored rectangle. A snapshot of the video at cue onset is also shown. The probability of gaze within a specific object increased after the person in the video pointed at the object, demonstrating a social cueing effect on eye movements. (b) Example gaze data from one observer within social cue events highlighted. Using a similar approach as that used for the analysis of SRTs to simple onsets, the latency of the first eye movement (i.e., point of gaze) towards socially referred object was extracted for all observers and all cue‐target events. Corresponding data for other videos are provided in Supporting materials.

We next conducted further exploratory analyses to examine whether differences in the timing of eye movements extend from simple saccadic responses to the onset of pictures to more complex eye movements in response to cues present in a child's natural interactions within their social environment. During the last visit at the age of 3 years, the children in the Tzaneen cohort were shown videos in which an adult speaker addressed the child and then pointed at different spatially separated pictures of objects on the wall (Figure 3). This scenario mimics a natural, early‐life learning situation where the child follows the nonverbal cues provided by the adult (i.e., hand gestures and eye gaze) to identify objects of interest (Csibra and György 2009).

**FIGURE 4:**
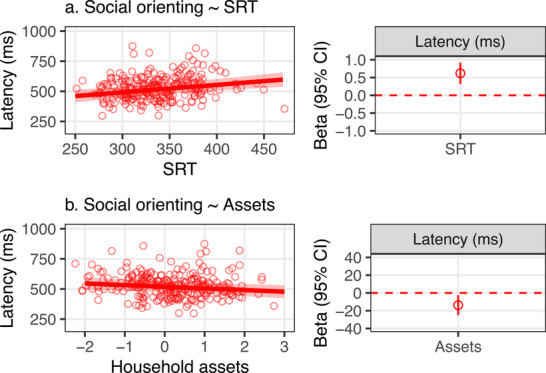
Latency to look toward a socially referred object was positively associated with SRT and negatively associated with the asset‐based resource index, with longer latencies observed in children who had longer SRTs or came from households with fewer resources.

Preliminary analyses of the eye tracking data collected during video viewing confirmed that children reacted to the pointing gestures in the predicted way; that is, looking at the referred objects increased after it had been pointed at (Figure 3). To quantify the latency of these responses, we used the same approach and rules for data processing as those used for the original analysis of SRTs to extract the latency from the onset of the cue to the first look at the referred object. SRTs to simple onsets and latencies to look towards the socially referred objects were directly related (β[95% CI] = 0.62 [0.32, 0.92], Figure 4). Also similar to the gradient in the SRTs to simple onsets, the latency to look towards the socially cued object varied along a gradient of household resources, with slower latencies in children from households with fewer resources (β[95% CI] = −13.74 ms [−25.02, −2.48], Figure 4). This gradient remained after the analyses were adjusted for prior exposure to TV (β[95% CI] = −12.15 ms [−23.53, −0.80]). Data availability (i.e., number of valid trials) was not correlated with SRT or household resources, *rs* < 0.10.

### 3.4 Other Aaspects of Early Development (South Africa)

Prior research has shown that multiple aspects of early development are associated with relative poverty in LMICs, including height and parent‐rated or expert‐evaluated cognitive and behavioral skills (Fernald et al. 2012; Fink et al. 2020; Rubio‐Codina et al. 2015; Nicolaou et al. 2020). For the current sample, longitudinal data were available for length/height (Visits 1–3), and parent‐rated skills or milestone achievements (Visits 1 and 2) (McCoy et al. 2016). Motor, language, and socioemotional skills were assessed at 36 months, using the Malawi Developmental Assessment Tool (MDAT, Gladstone et al. 2010). As predicted, child height‐for‐age *z*‐score (HAZ) was positively associated with household resources (β[95% CI] = 0.2 [0.08, 0.33]) with no clear age‐related differences in the association (Figure 2). Parent‐rated development also showed a trend for the expected gradient at 7 and 17 months (β[95% CI] = 0.07 [−0.01, 0.15]), with slightly clearer gradient at 17 months (Figure 1, these data were not available for the 36‐month assessments). Contrary to predictions, we observed little variation in the MDAT scores in the sample and no association between MDAT scores and household resources.

### 3.5 Replication (Zambia)

In Zambia, eye movement data were collected at the post‐intervention visit at the mean age of 31 months, HAZ at the pre‐ and post‐intervention visits, and assessments of motor, cognitive, and language skills at the post‐intervention visit. The methods employed for assessing SRTs and child development and the items in the assent index in the Zambian study were slightly different from those in the South African study (Supporting Methods), but were designed to address the same underlying constructs.

**FIGURE 5:**
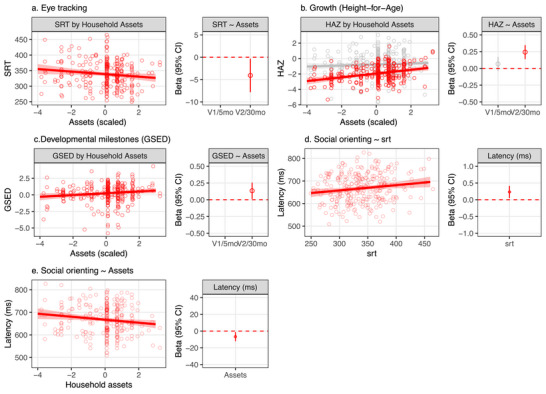
Child development measures in a replication analysis of data collected in Zambia. (a) Observed and predicted values of SRT by household asset ownership and beta coefficients for household assets from a linear mixed effects model with the SRT as a dependent variable and household assets as a predictor. (b, c) Corresponding data for HAZ and an aggregate measure of observed motor, cognitive, and language skills (Global Scale of Early Development, GSED). (d, e) Corresponding data for the latency to look at socially cued object as a function of SRT and household assets.

The results from the South Africa sample were largely replicated in the Zambia cohort data analyses (Figure 5). Eighty‐seven percent of children assessed with eye tracking in Zambia were retained in the analyses, and mean SRTs as well as reliability estimates (Split‐half *rs*) were comparable to those in the primary analyses (Table 2). Longer SRTs were found in children from households with fewer assets, although the gradient was shallower than that observed in the South African cohort β[95% CI] = −4.07 ms [−7.83, −0.32], Table S4). The association between SRT and household assets remained marginal when the model was adjusted for screen time (β[95% CI] = −3.42 ms [−7.15, 0.30]) and negative but nonsignificant when adding HAZ (β[95% CI] = −2.69 ms [−6.55, 1.18]). As found in the South Africa sample, SRT was inversely associated with HAZ in the Zambia cohort (β[95% CI] = −6.31 ms [−11.24, −1.40]).

The latency to look towards the socially cued object was directly associated with SRT, β[95% CI] = 0.23 ms [0.05, 0.42], and inversely associated with asset‐based resources, with slower latencies in children from households with fewer asset‐based resources (unadjusted β[95% CI] = −6.66 ms [−12.14, −1.19], adjusted for screen time, β[95% CI] = −6.53 ms [−12.02, −1.00]). HAZ was not associated with household resources at the first visit at the average age of 5 months, but a positive association was observed at the second visit when children were 31 months with lower HAZ in children from households with fewer asset‐based resources (β[95% CI] = 0.24 [0.14, 0.35]). Scores in GSED were associated with household asset index with lower scores in children from households with fewer asset‐based resources (β[95% CI] = 0.13 [0.01, 0.26]).

## 4 Discussion

Our results demonstrate individual differences in the timing of saccadic eye movements in infants, a behavior that is fundamental to early visual exploration and learning (Robertson et al. 2012) and also a salient cue in social signaling (Foulsham 2015; Pfeiffer et al. 2012). The differences in the timing of eye movements co‐varied with socioeconomic characteristics of the early environment, as assessed by household assets. This result provides a critical expansion to prior studies focusing on children's physical growth or relying on coarser or more global measures of young children's behavioral competencies (e.g., parent reports) in low‐resource settings (Fernald et al. 2012; McCoy et al. 2016) or studies looking at gradients in the timing of eye movements across income or parent‐education levels in high‐resource settings (Conejero and Rueda 2018).

The magnitude of the association between SRT and household resources increased by age, being most pronounced at the last, 36‐month visit. This result may indicate a true change in the magnitude of the association and suggest that the cognitive trajectories in infants from different socioeconomic strata become increasingly more diverged as the children grow older. However, differences in the magnitude of the associations between visits are, at present, not straightforward to interpret, as they may also arise from improvements in the technical quality of the SRT measurements at older ages (i.e., higher signal‐to‐noise ratio of the SRT estimates and associated correlations) (Räsänen et al. 2023). Further analyses are, therefore, necessary to assert that the age differences are not explained by higher levels of measurement noise at 7 and 17 months.

The estimated difference in SRT between infants from households with the highest and lowest levels of resources may not appear large (∼50 ms), but a difference of this magnitude may still have significant implications on visual exploration and social signaling. Even within the short assessment conducted in the current study, an average delay of 50 ms in relocating the point of gaze (i.e., sharp foveal vision) to the direction of a new visual stimulus translates into a 2‐s difference in the total time spent in foveal processing of the pictures shown. The association between SRT and latency of orienting to social cues suggest that the documented individual differences may generalize across contexts involving visual exploration and possibly scale up quickly in naturalistic situations that demand frequent shifts of the location of visual attention. As a salient social signal about the locus of one's attention, variations in speed may also have implications for social interactions (Pfeiffer et al. 2012).

A key unaddressed question concerns the mechanisms that mediate the demonstrated covariance between eye movement times and early socioeconomic environment. This study analyzed data collected in predominantly resource‐poor settings where household asset ownership is best interpreted as a proxy measure for the relative poverty of the child's rearing environment. Differences in child development may reflect both genetic and environmental factors that are correlated with SES (Krapohl and Plomin 2016), but in low‐resource settings, individual differences in cognition are less likely to reflect genetic than environmental effects (Tucker‐Drob et al. 2011). Household asset ownership in these settings may serve as a proxy for multiple influential factors, including nutrition and levels of sensory and social stimulation (Hair et al. 2016; McCormick et al. 2020). SRT is correlated with the nutritional status of the child, as suggested by our current findings showing longer SRTs in children with lower HAZ (a marker of chronic nutritional status), and by previous results showing that SRTs can be positively influenced by nutritional supplementation during recovery from acute malnutrition (Leppänen et al. 2022) or by supplementation provided to the mother during pregnancy (Caudill et al. 2018). However, our analyses provided partial evidence that the gradient in SRT persisted even after including HAZ as a covariate in the model, suggesting that it is also influenced by factors beyond the child's chronic nutritional status.

Further research is needed to examine whether differences in SRT are related to other household‐level variations that correlate with asset‐based resources but were not assessed in this study. Our data suggest that these differences were not explained by screen time, although we did replicate previous findings showing that SRT is related to screen time (Portugal et al. 2021). However, we did not assess other aspects of the children's sensory and social environments that may be influential, such as possible variations in the quantity and quality of dyadic infant–parent interactions. During early stage of development, dyadic interaction between the child and the parent rely heavily on nonverbal gestures, and the quantity of these gesture correlates positively with socioeconomic status (Rowe and Goldin‐Meadow 2009).

The possibility that the development of eye movements is influenced by exposure to dyadic interactions, gestures, and other spatial cues is plausible as various laboratory experiments suggest that different aspects of eye movements are sensitive to activity‐dependent plasticity. This includes the adjustment of saccade amplitude over repeated trials (Alahyane et al. 2016), as well as training‐related improvements that transfer across saccade axes (e.g., from horizontal to vertical saccades) and saccade types (e.g., from reflexive to voluntary saccades) (Montenegro and Edelman 2019). During development, plasticity may occur in different components of the network that underlies saccadic eye movements, including visual areas, which tend to mature early, as well as associative areas in the parietal and frontal regions, which follow a more protracted developmental time course (Lee et al. 2024).

## Author Contributions

P.C.R., J.M.L., J.P., G.F., D.E., L.C., D.P., A.K.Y., and D.H.H. designed the study. D.E., L.C., and J.P. provided daily oversight, enrolment, and data entry. J.M.L., J.P., and P.C.R. curated the data and performed statistical analysis. All authors interpreted the data. J.M.L. wrote the first draft of the manuscript. All authors read and approved the final manuscript.

## Conflicts of Interest

The authors declare no conflicts of interest.

## Supporting information


**Supporting File 1**: desc70058‐sup‐0001‐SupMat.pdf

## Data Availability

The data that support the findings of this study are available on request from the corresponding author.
