# Supplementary material for: Early Emerging Gradients in Children's Eye Movement Times Across Levels of Household Resources
Source: Dev Sci. 2025 Aug 12;28(5):e70058. doi: 10.1111/desc.70058 (PMC12344327; doi:10.1111/desc.70058)
Supplement: Supplementary file 1 — Supporting File 1: desc70058‐sup‐0001‐SupMat.pdf [file DESC-28-e70058-s001.pdf]

Supplementary Materials for "Early-Emerging Gradients in Children's Eye Movement Times Across Levels of Household Resources"

J.M. Leppänen[1], J. Pyykkö[2], D. Evans[3], L. Coetzee[3], G. Fink[4], A.K. Yousafzai[5], D.H. Hamer[2,6], J.D. Parkerson[7],& P.C. Rockers[2]

[1]Department of Psychology and Speech-Language Pathology, University of Turku, Turku, Finland

[2]Department of Global Health, Boston University, Boston, MA, USA

[3]Health Economics and Epidemiology Research Office, Department of Internal Medicine, School of Clinical Medicine, Faculty of Health Sciences, University of the Witwatersrand, Johannesburg, South Africa

[4]Swiss Tropical and Public Health Institute, University of Basel, Allschwil, Switzerland

[5]Department of Global Health and Population, Harvard T.H. Chan School of Public Health, Boston, MA, USA

[6]Section of Infectious Diseases, Department of Medicine, Boston University School of Medicine, Boston, MA, USA

[7]Innovations for Poverty Action, New York, NY, USA

Supplementary methods

Eye tracking assessments

Setting and equipment

Eye-tracking measurements were conducted in a dedicated room following the procedures described in ref. (Leppänen et al., 2022). A partition was used to divide the room into separate spaces for the data collector and the participant. During the testing, participants were presented with short series of visual and auditory stimuli on a 30 x 30cm (South Africa) or 53 x 30cm (Zambia) computer screen (refresh rate: 60 Hz). The participant's point of gaze was recorded at 120 samples/sec with a Tobii X3-120 (South Africa) or Tobii Pro Fusion eye tracker (Zambia). The presentation of the stimuli and the synchronization of the stimulus event and eye tracking data were managed by custom Python scripts (https://github.com/infant-cognition-turku/ldrop) or Titta toolbox (Niehorster et al., 2020), Psychopy functions (Peirce, 2007) and a Tobii SDK plug-in, running on Linux Mint 17 (South Africa) or Windows 11 Pro (Zambia) operating system. The computer and the computer screen were connected with a HDMI-DVI cable. The computer and the eye tracker were connected via an Ethernet cable and a TRENDnet 4-Port Broadband Router (TRENDnet, Inc. Torrance, CA) or a USB-C port.

Procedure and stimuli

Data collectors who were fluent in local languages in South Africa (Tsonga/Sepedi) or Zambia (Nyanja/Bemba) were trained to perform the eye tracking assessments. Two data collectors and the child's caregiver were typically present in the assessment of a child. The caregiver held the child in a forward-facing baby carrier or in their lap during the assessment and were seated so that the child's eyes were at a 60 cm distance from the eye tracker and at an optimal height in relation to the tracking "box" of the eye tracker. To ensure that the child's and not the caregiver's eyes were tracked, the caregiver was instructed to turn their

head and eyes to the side (∼90 degrees from the screen) and to avoid looking at the screen during the assessment. After the optimal position for the child was found, the data collector moved behind a partition for the duration of testing, but kept monitoring the child's position through online visualization of the tracked position of the child's head/eyes with respect to the optimal tracking position.

The test started with calibration, followed by the presentations of short videos of social scenes and saccade targets. Videos and saccade targets were presented in alternating sequence to minimize the monotony of the test sessions. In South Africa, the order of the stimuli was as follows: 1) three to nine calibration targets, 2) a long social scene, 3) five saccade targets, 4) a short social scene, 5) five saccade targets, 6) a long social scene, 7) five saccade targets, 8) a short social scene, and 9) five saccade targets. Children completed this sequence twice, thus seeing a total of 6-18 calibration targets, 8 blocks of 5 saccade targets (40 saccade targets in total), and 4 long and 4 short social scenes on each visit. In Zambia, the assessments was started with 5 to 15 calibration and calibration validation targets (5+4 per attempt), followed by six blocks, each consisting of a video and 11 saccade targets (6 videos and 66 saccade trials in total).

If the eye tracking system lost contact with the child's eyes or the child became restless, inattentive or fussy during the assessment, the data collector administered a break in the testing and performed required adjustments (e.g., adjusted the caregiver's and the child's position).

Calibration targets

In South Africa, the calibration targets were white discs (1.3°x 1.3°) presented in the center and all corners of the screen after an animation had first been displayed in each location to attract the child's gaze. Gaze points that fell within a 9°x 9° rectangle surrounding the target were used in calibration, which was based on a similarity transformation estimator (https://github.com/axelpale/nudged). Calibration accuracy was validated by calculating

the mean distance of raw and calibrated gaze points to the targets presented at the start of the assessment and again half-way through the assessment. In Zambia, the calibration target was a white fixation cross with a small black dot in the center (Thaler et al., 2013) presented in five locations for calibration and four new location for calibration validation. Validation results including accuracy, precision, and data loss were presented and visualized to the data collector. If satisfactory validation results (∼1 deg) were not achieved on the first attempt, the calibration and validation process was repeated up to two times. Data on calibration accuracy is given in Supplementary Table 2. No participant was excluded on the basis of the calibration outcome alone in the present analysis as the quality control for valid SRTs automatically excludes trials on which the calibration was not at sufficient level for reliable determination of SRT. Observers lacking sufficient number of valid SRT trials on a visit were automatically excluded.

Saccade targets

In South Africa, saccade targets were colored animated cartoon drawings of objects (e.g., bird, mouse, face, fish, pig, or soccer ball, size 5.7° x 5.7°, Supplementary Figure 1). The animation remained paused at the first frame and the accompanying audio silenced until the child looked at it (i.e., a gaze point overlapping with the target area was recorded) or a 1 second timeout value passed. The animation was then played for 2500 msec with an accompanying sound (e.g., bird singing). In Zambia, saccade targets were sinusoidal grating stimuli with a Gaussian mask (size 5.0° x 5.0°, Supplementary Figure 1). For a randomly chosen subset of the targets, an animated cartoon was superimposed on the grating after a delay to maintain the child's interest in the task. Following a previous study (Leppänen et al., 2022), the first target was presented in the center of the screen (0°,0°), and each subsequent target in a new randomly chosen on-screen location 10° (South Africa) or 8°-14° away (Zambia). The first target in the center of the screen was excluded from the analysis as the position of the gaze at the onset of the target and, consequently, the starting position of the

first saccade was not standardized. Following the first target children saw 5 (South Africa) or 11 (Zambia) new targets in one block, and 8 (South Africa) or 6 (Zambia) blocks in total. The targets and the locations were selected randomly from two pre-existing, randomized lists (South Africa) or from a unique list of random locations generated separately for each block and assessment (Zambia).

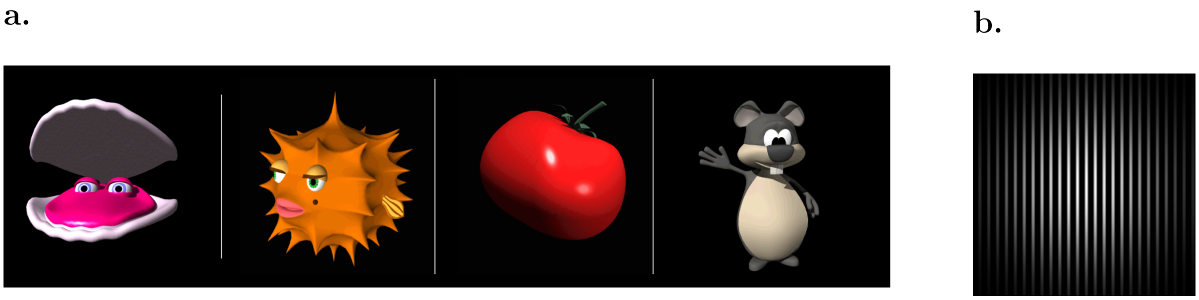

Supplementary Figure 1: Examples of saccade targets. Colored cartoon drawings were used as saccade targets in the study conducted in South Africa (a). A sinusoidal grating stimulus with a Gaussian mask was used in the study conducted in Zambia (b).

Social scenes

Social scenes used in the South Africa study were 4- to 47-second video recordings of two South African models. One of the models spoke Tsonga and the other Sepedi, the two languages spoken in the study population. Half of the videos were short 4 to 8-second clips in which the models looked directly at the camera and greeted the child. The other half of the videos were longer 12 to 45-sec video recordings of dyadic (i.e., child-adult) or triadic (child, adult and an object or child and two adults) interaction scenarios in which the model enacted a short story while looking at the camera, or while alternating looks between the camera and an object/another person (e.g., fruits that the model was holding in their hand, pictures on the wall, or another person appearing in the video). The videos were taken against a variable background in an office environment. Children saw a total of 4 short videos and 4 long videos (1 presentation per video) on each visit. The videos were of both

the Tsonga and Sepedi speaking models (7-month-visit) or only of the model speaking the child's native language (17- and 36-month visit). The aspect ratios of the scenes used at the 7- and 17-month-visit were inadvertently distorted. This error was corrected for the 36-month assessment (data used in the current study). Similar videos were used in the study in Zambia with the difference that the scenes depicted a different set of activities.

Supplementary data analyses and results

SRT

Correlations between Mean SRT and the number of valid trials and calibration outcome (distance to the real location of the target in degrees of visual angle) are shown in Supplementary Table 1. No systematic dependency between mean SRT and data quality indicators were found at either sites.

Supplementary Table 1: Associations (Pearson r [95% CI]) of eye tracking data quality measures with the dependent variables, household wealth, and screen time.

Variable	M (SD)	SRT	Wealth	Screen time
			Tzaneen	
Trials v1/7m	18.74 (6.70)	-0.15 [-0.30 -0.01]	0.10 [-0.05 0.25]	-0.00 [-0.15 0.15]
Trials v2/17m	20.73 (6.73)	-0.09 [-0.21 0.03]	0.02 [-0.10 0.15]	-0.12 [-0.25 0.01]
Trials v3/36m	25.15 (6.09)	-0.02 [-0.14 0.11]	0.00 [-0.12 0.13]	0.05 [-0.08 0.17]
Calibr. v1/7m	0.94 (0.40)	-0.13 [-0.28 0.03]	-0.11 [-0.26 0.05]	-0.12 [-0.27 0.04]
Calibr. v2/17m	0.60 (0.32)	-0.07 [-0.21 0.09]	0.03 [-0.12 0.18]	-0.07 [-0.23 0.09]
Calibr. v3/36m	0.48 (0.20)	-0.00 [-0.16 0.16]	0.10 [-0.06 0.25]	-0.09 [-0.25 0.06]
			Lusaka	
Trials v2/30m	22.43 (8.57)	-0.04 [-0.16 0.08]	-0.03 [-0.15 0.09]	0.02 [-0.09 0.14]
Calibr. v2/30m	1.32 (0.61)	0.12 [-0.00 0.24]	-0.06 [-0.18 0.06]	-0.05 [-0.17 0.07]

Latency of eye movements towards socially cued objects.

Supplementary Figures 2-13 visualize eye movement responses to socially cued objects. The figures show the mean probability (95% CI) of gaze within different object areas as a function of video time (a). The 1-sec period following a cue towards the object is highlighted with

a colored rectangle. A snapshot of the video at cue onset is also shown. The probability of gaze within a specific object was generally low, but increased distinctively after the person in the video pointed at the object, demonstrating a social cueing effect on eye movements. The figures also show example gaze data from one observer with the sections involving a social cue as well as the latency of the oberver's response highlighted (b). Using a similar approach, the latency of the first eye movement (i.e., point of gaze) towards socially referred object after the cue was extracted for all observers and all cue-target events.

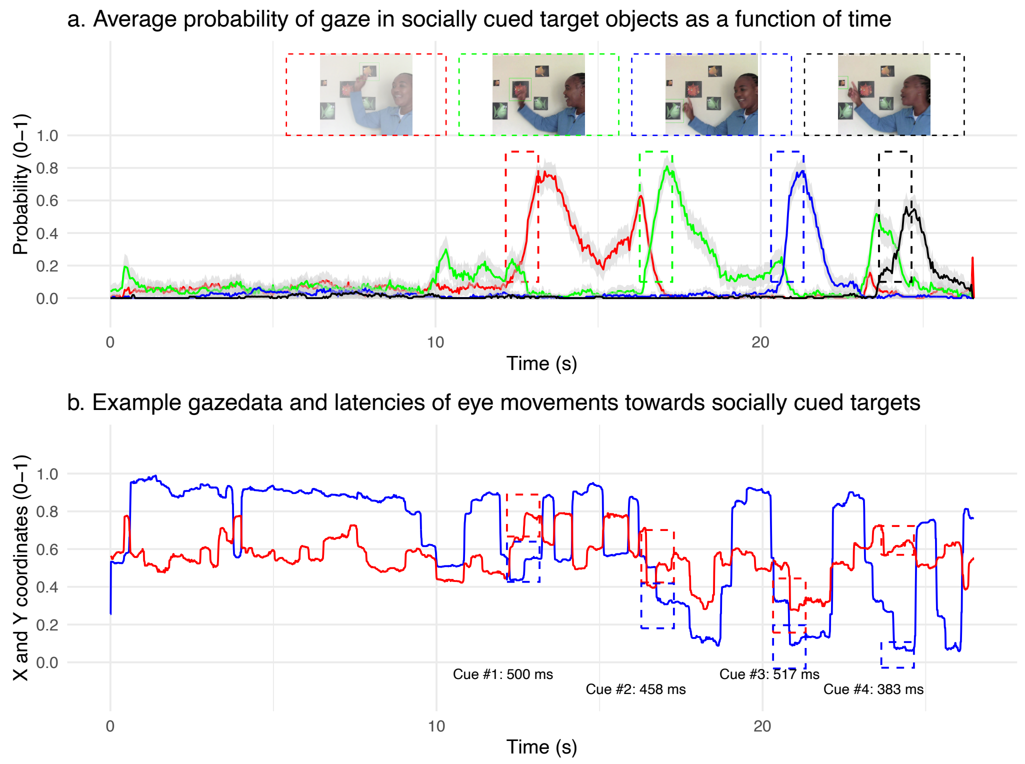

Supplementary Figure 2: South Africa, video 1 (language: Tsonga)

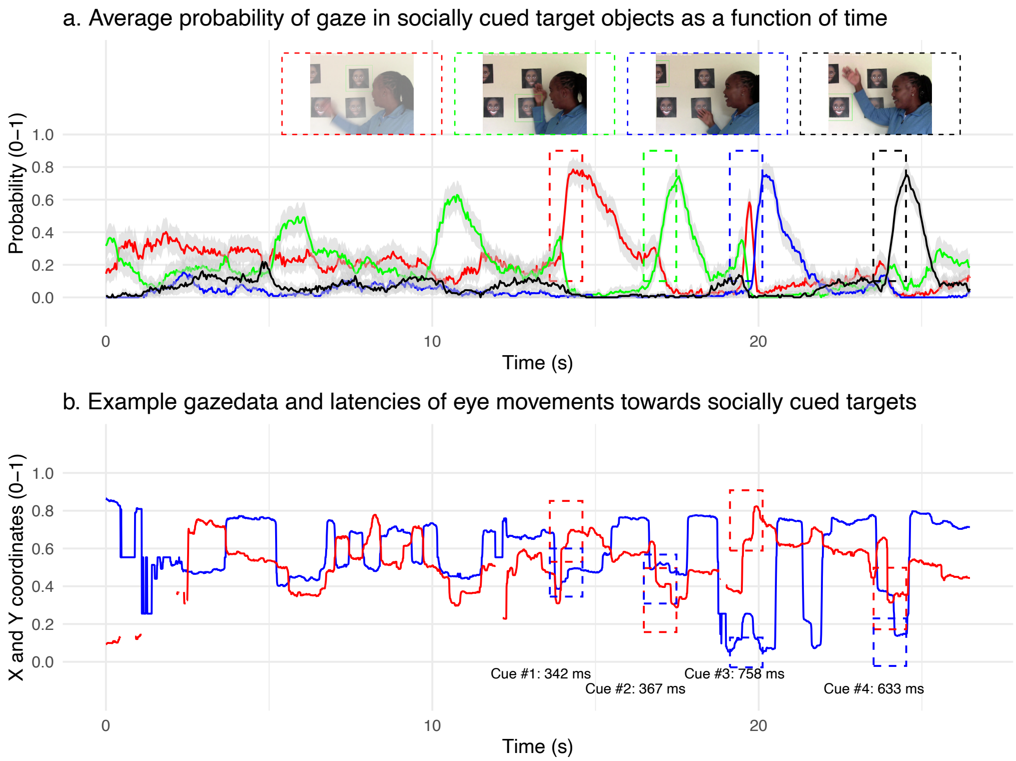

Supplementary Figure 3: South Africa, video 2 (language: Tsonga)

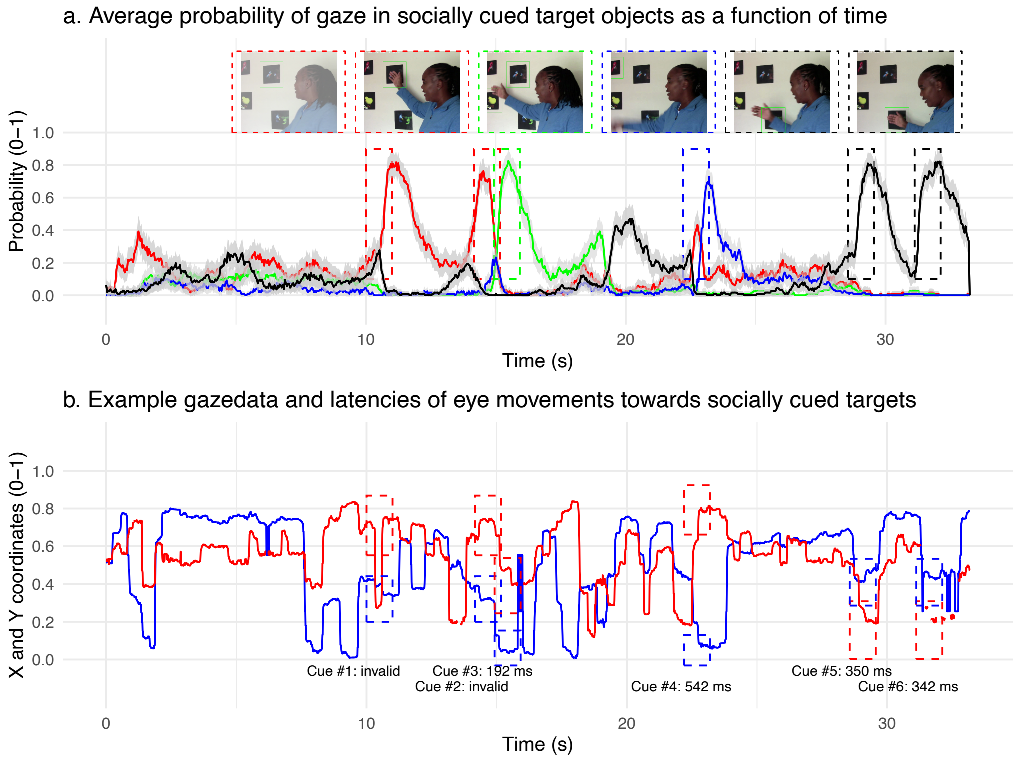

Supplementary Figure 4: South Africa, video 3 (language: Tsonga)

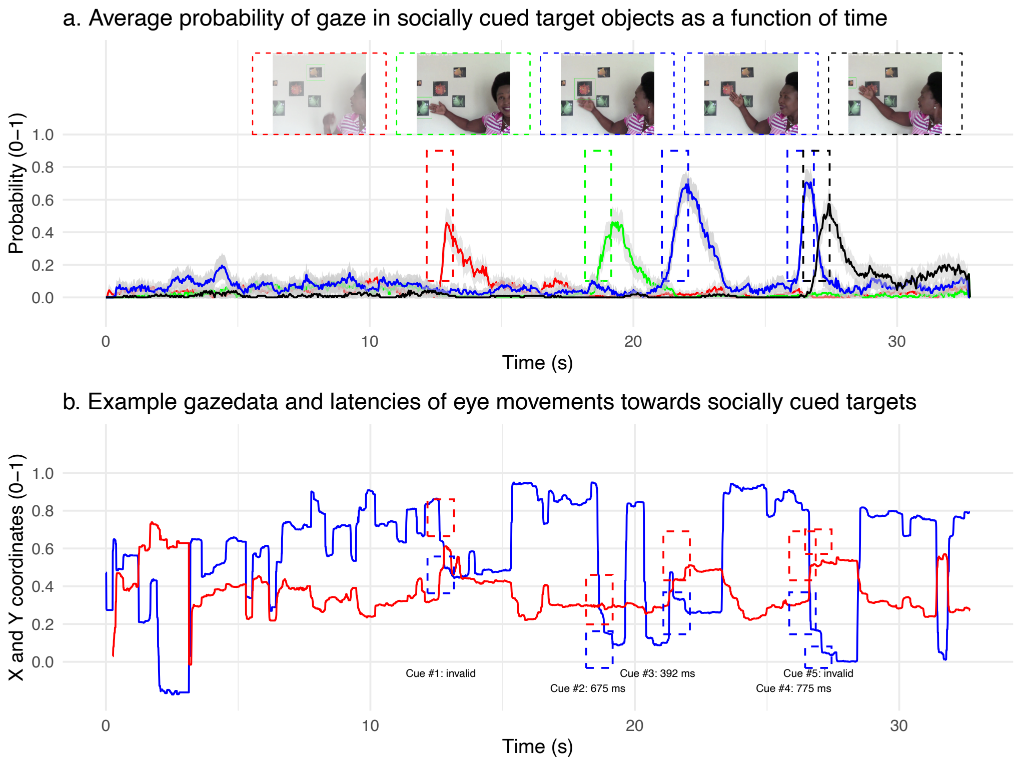

Supplementary Figure 5: South Africa, video 1 (language: Sepedi)

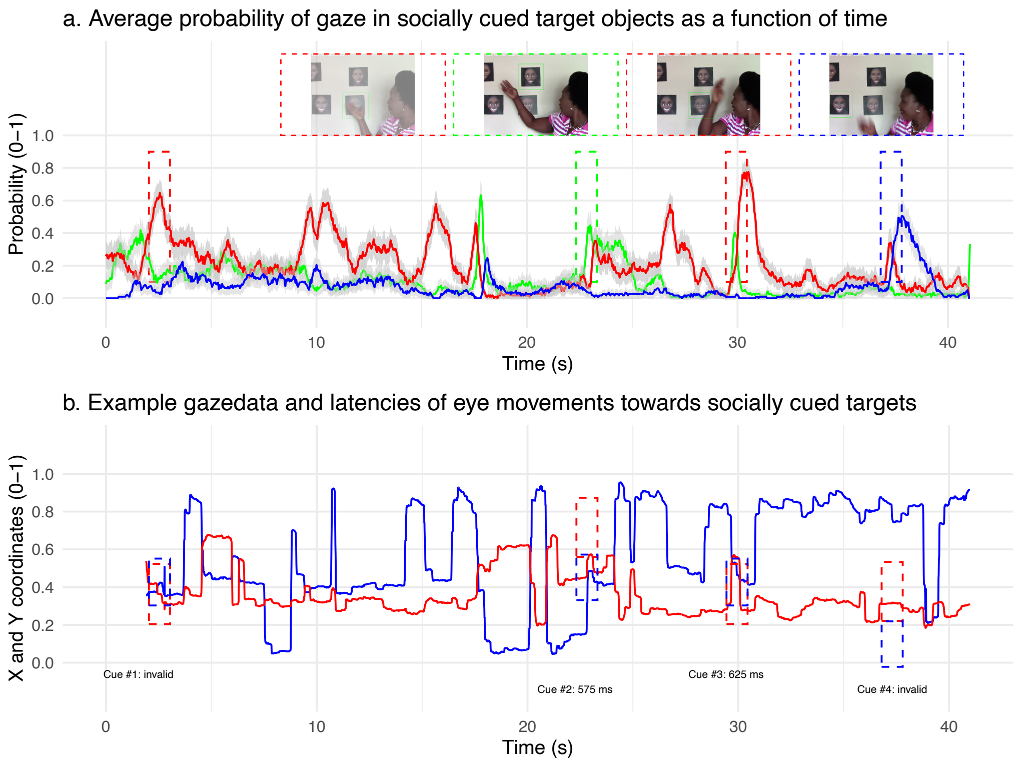

Supplementary Figure 6: South Africa, video 2 (language: Spedi)

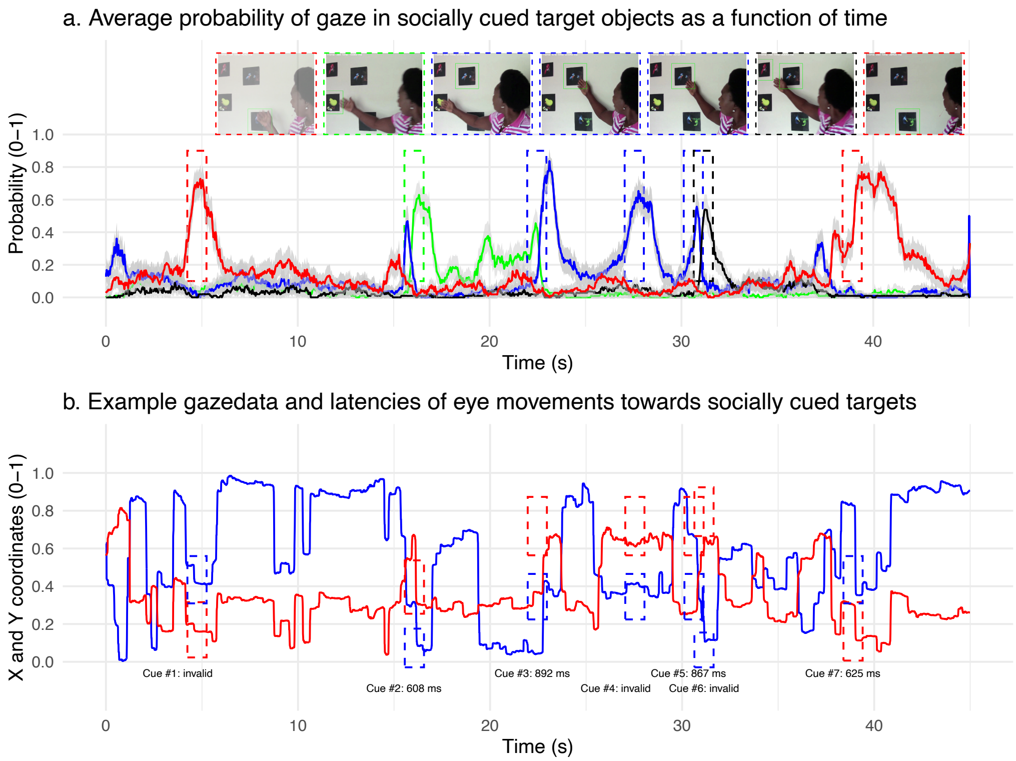

Supplementary Figure 7: South Africa, video 3 (language: Sepedi)

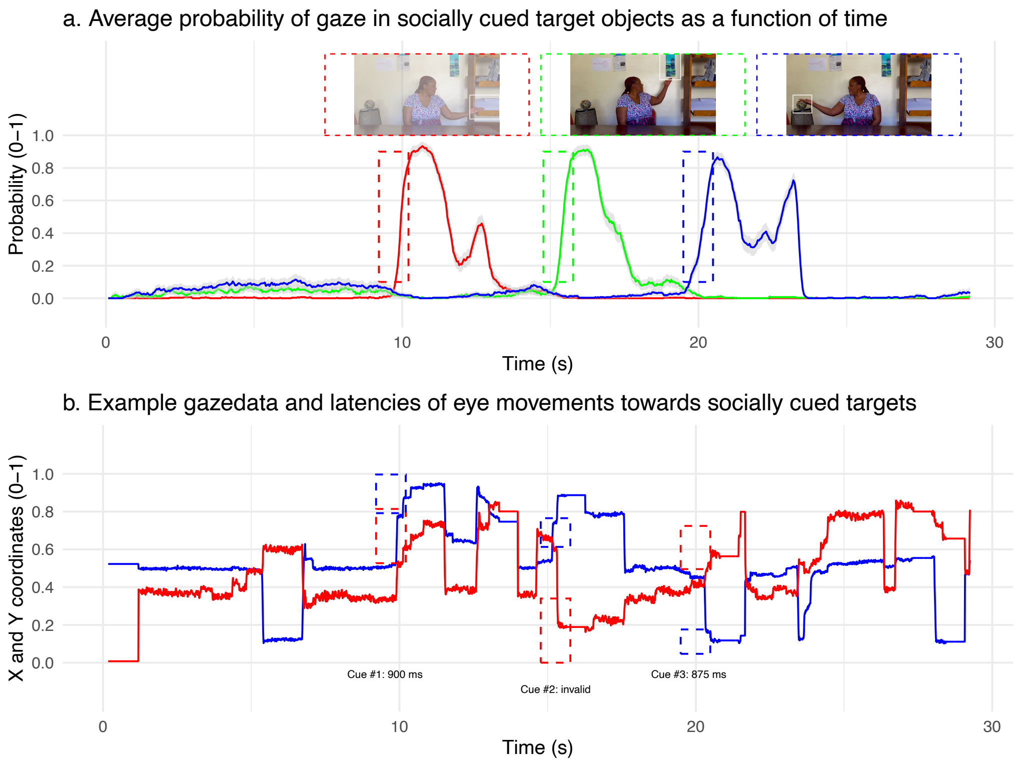

Supplementary Figure 8: Zambia, video 1

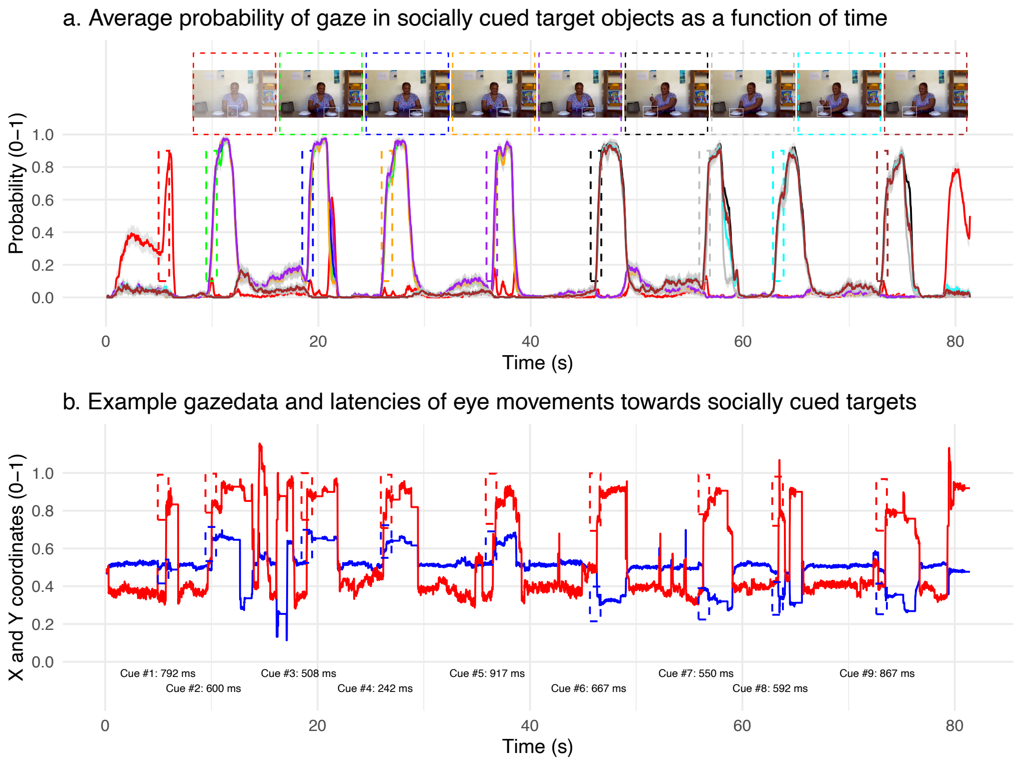

Supplementary Figure 9: Zambia, video 2

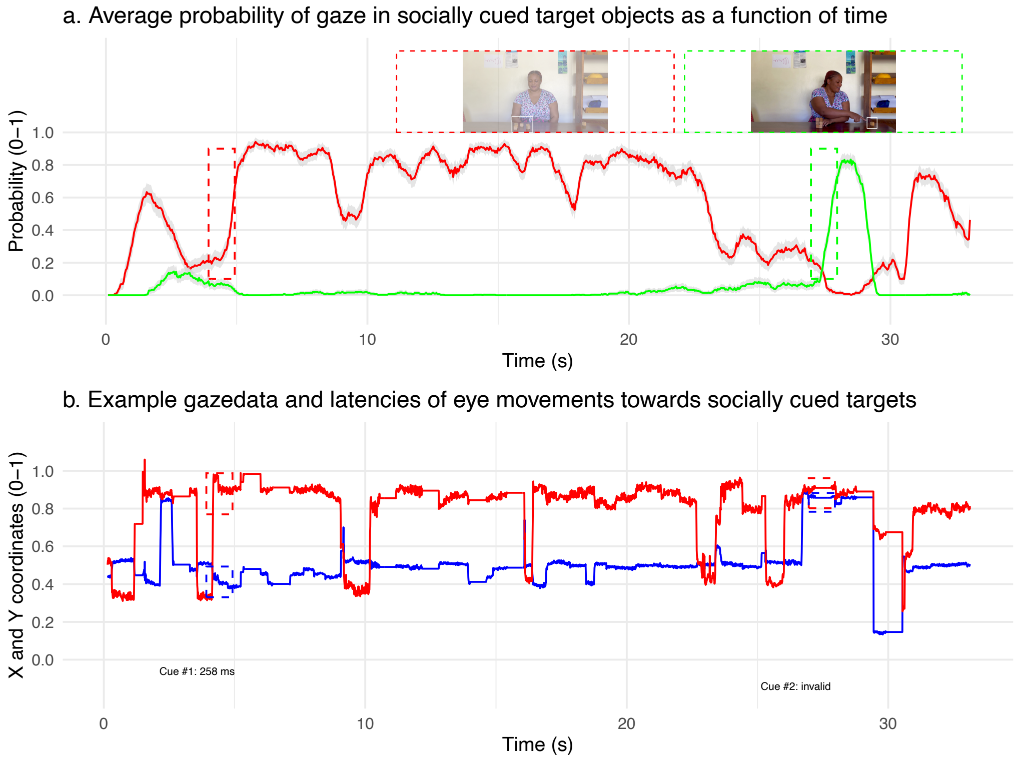

Supplementary Figure 10: Zambia, video 3

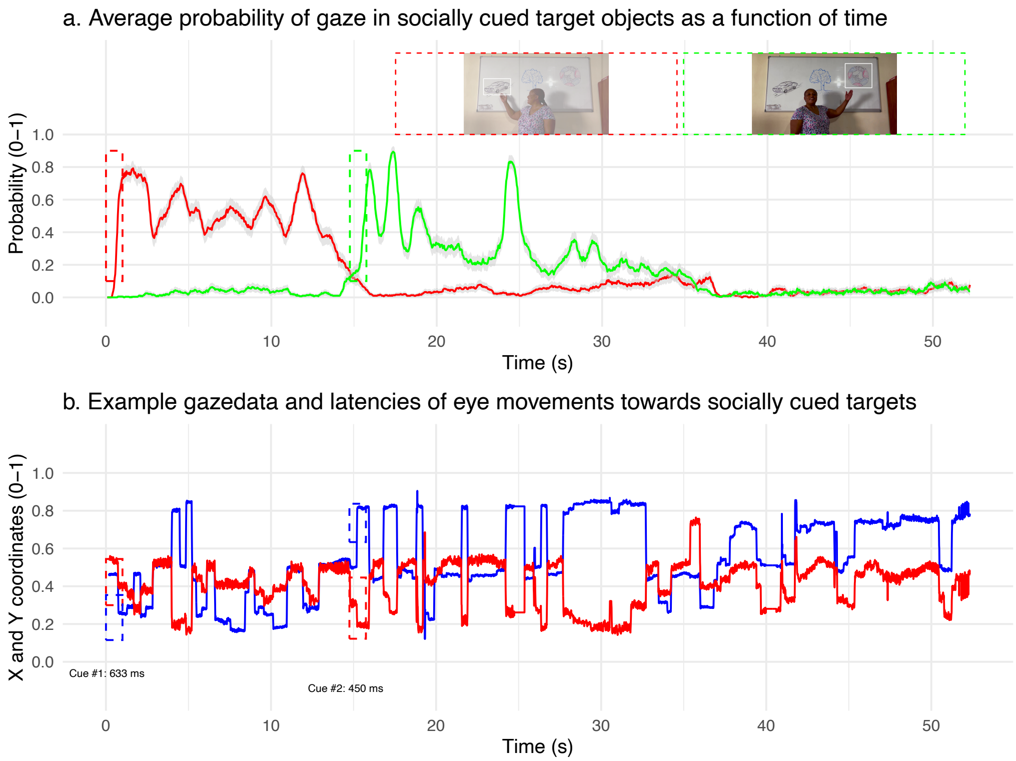

Supplementary Figure 11: Zambia, video 4

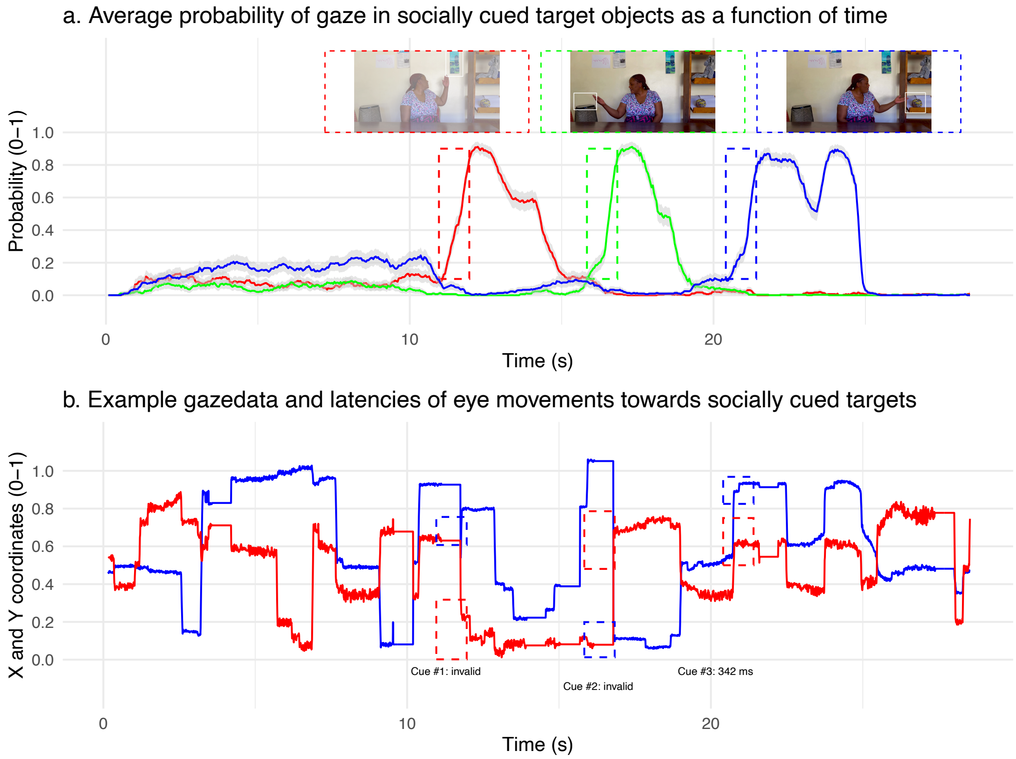

Supplementary Figure 12: Zambia, video 5

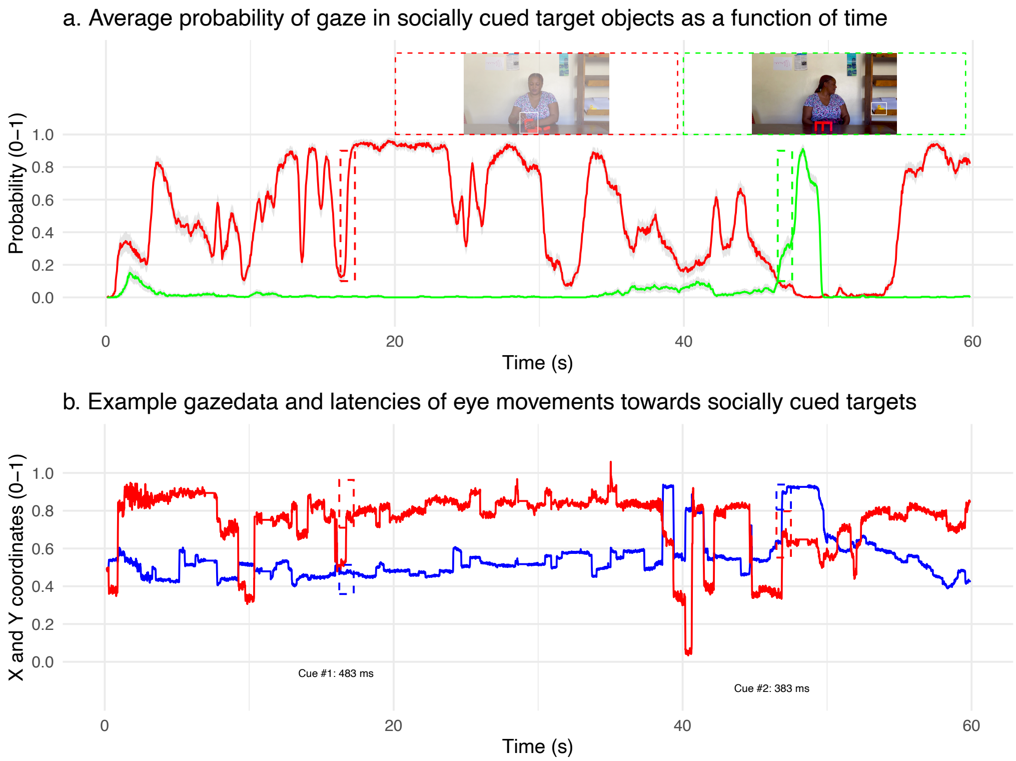

Supplementary Figure 13: Zambia, video 6

Supplementary analyses of the association between eye movement measures and household wealth

Correlations between data quality indicators and household wealth are shown in Supplementary Table 1. Full results of linear mixed models with the eye tracking outcome measure as the response variable and household wealth as a predictor are provided in Supplementary Tables 2 -5. Participants in the South Africa cohort were a subsample of caregiver-child dyads enrolled in a cluster-randomized controlled trial that was designed to evaluate the impact of a package of early childhood interventions in Mopani District (SouhSANCTR registration number: PACTR201710002683810). Participants in the Zambia cohort were a subsample of children in a study on the effects of a growth chart intervention and nutritional supplement on child growth (clinicatrials.gov registration number: NCT05120427). The wealth effects shown in Supplementary Tables 2-5 were largely similar when a variable representing treatment status was added to the model as a fixed effects covariate.

Supplementary Table 2: Coefficients from a linear mixed model predicting the latency of saccadic eye movement responses by household wealth in the South Africa cohort (SRT∼Visit x Wealth + Age at visit + Household size + (1|Participant) + (1|Block:trial number))

Fixed effect	Estimate	95 % CI	Z	P >|Z|
Intercept	379.84	368.26 – 391.41	65.44	<.001
Visit [2]	-9.34	-12.69 – -5.98	-5.45	<.001
Visit [3]	-55.24	-58.61 – -51.87	-32.13	<.001
Wealth	-5.43	-9.17 – -1.69	-2.85	0.004
Household size	2.27	0.94 – 3.59	3.36	0.001
Age at visit	-0.01	-0.04 – 0.02	-0.54	0.588
Visit [2]: Wealth	-0.9	-3.95 – 2.15	-0.58	0.561
Visit [3]: Wealth	-5.35	-8.5 – -2.2	-3.33	0.001
Random Effects				
σ^2	5130.81			
τ_{00} Participant	829.96			
τ_{00} Block	75.3			
τ_{00} Block:trial	70.61			
N Participant	371			
Observations	15510			
Marginal R^2	0.11			

Supplementary Table 3: Coefficients from a linear mixed model predicting the latency of eye movements towards socially cued targets by household wealth in the South Africa cohort (Social orienting latency ∼ Wealth + Household size + Age at visit + (1|Participant) + (1|Cue))

Fixed effect	Estimate	95 % CI	Z	P >|Z|
Intercept	543.21	473.01 – 613.3	15.48	<.001
Wealth	-13.74	-25.02 – -2.48	-2.4	0.016
Household size	-0.23	-6.83 – 3.16	-0.72	0.469
Age at visit	-0.23	-0.37 – -0.09	-3.29	0.001
Language	-25.43	-116.99 – 66.07	-0.56	0.574
Random Effects				
σ^2	32509.43			
τ_{00} Participant	3592.01			
τ_{00} Cue	14311.41			
N Participant	240			
Observations	2108			
Marginal R^2	0.01			

Supplementary Table 4: Coefficients from a linear mixed model predicting the latency of saccadic eye movement responses by household wealth in the Zambia cohort (SRT∼Wealth + Household size + Age at visit + (1|Family) + (1|Block) + (1|Target location))

Fixed effect	Estimate	95 % CI	Z	P >|Z|
Intercept	344.81	262.96 – 426.77	8.23	<.001
Wealth	-4.07	-7.83 – -0.32	-2.12	0.034
Household size	1.16	-0.66 – 2.99	1.24	0.213
Age at visit	-0.44	-3.10 – 2.21	-0.33	0.743
Random Effects				
σ^2	5719.71			
τ_{00} Family	1428.78			
τ_{00} Block	10.70			
τ_{00} Target location	165.32			
N families	265			
Observations	6168			
Marginal R^2	0.005			

Supplementary Table 5: Coefficients from a linear mixed model predicting the latency of eye movements towards socially cued targets by household wealth in the Zambia cohort (Social orienting latency ∼ Wealth + Household size+ Age at visit + (1|Family))

Fixed effect	Estimate	95 % CI	Z	P >|Z|
Intercept	718.27	599.06 – 837.48	11.77	<.001
Wealth	-6.66	-12.14 – -1.19	-2.38	0.017
Household size	-1.7	-4.42 – 1.03	-1.21	0.224
Age at visit	-1.3	-5.14 – 2.54	-0.66	0.509
Random Effects				
σ^2	40558.52			
τ_{00} Participant	857.31			
N Families	296			
Observations	3805			
Marginal R^2	0.003			

References

Leppänen, J. M., Butcher, J. W., Godbout, C., Stephenson, K., Hendrixson, D. T., Griswold, S., Rogers, B. L., Webb, P., Koroma, A. S., & Manary, M. J. (2022). Assessing infant cognition in field settings using eye-tracking: a pilot cohort trial in Sierra Leone. *BMJ Open*, *12*(2), e049783.

Niehorster, D., Andersson, R., & Nyström, M. (2020). Titta: A toolbox for creating psychtoolbox and psychopy experiments with tobii eye trackers. *Behavior Research Methods*, *52*. https://doi.org/10.3758/s13428-020-01358-8

Peirce, J. W. (2007). PsychoPy–Psychophysics software in Python. *J Neurosci Methods*, *162*(1-2), 8–13.

Thaler, L., Schütz, A. C., Goodale, M. A., & Gegenfurtner, K. R. (2013). What is the best fixation target? The effect of target shape on stability of fixational eye movements. *Vision Research*, *76*, 31–42. https://doi.org/10.1016/j.visres.2012.10.012
